# Levator muscle changes in Marcus Gunn jaw-winking syndrome and other forms of ocular congenital cranial dysinnervation disorder

**DOI:** 10.5935/0004-2749.2024-0236

**Published:** 2025-04-07

**Authors:** Hind Manaa Alkatan, Nawaf Alkuhaimi, Adel H. Alsuhaibani, Azza M. Y. Maktabi

**Affiliations:** 1 Department of Ophthalmology, College of Medicine, King Saud University, Riyadh, Saudi Arabia; 2 King Saud University Medical City, College of Medicine, King Saud University, Riyadh, Saudi Arabia; 3 Pathology Department, College of Medicine, King Saud University, Riyadh, Saudi Arabia; 4 Pathology and Laboratory Medicine Department, King Khaled Eye Specialist Hospital, Riyadh, Saudi Arabia

**Keywords:** Blepharoptosis/congenital, Cranial nerves/abnor-malities, Blepharoptosis/surgery, Ocular muscles, Synkinesis, Eyelid diseases, Ocular motility disorders/surgery, Ophthalmologic surgical procedures

## Abstract

**Purpose:**

This study was conducted to report the histopathological and clinical features of the Marcus Gunn phenomenon and other similar conditions of upper eyelid misfiring.

**Methods:**

This was a retrospective study of patients with congenital ptosis with Marcus Gunn phenomenon who have undergone surgical repair over a period of 12 years and another two patients with upper eyelid misfiring in association with extraocular movements to identify their histopathological findings as subtypes representing ocular congenital cranial dysinnervation disorder.

**Results:**

Among 136 patients with congenital ptosis, 11 (8%) patients with Marcus Gunn phenomenon or misfiring were identified, of whom 9 (6.6%) had typical known Marcus Gunn phenomenon and 2 (1.4%) had eyelid misfiring similar to Marcus Gunn phenomenon. In all patients, the histopathological changes of the excised levator muscle included overall loss and/or atrophy of muscle fibers and irregular-modified Gomori trichrome staining.

**Conclusion:**

The Marcus Gunn phenomenon and similar misfiring conditions with synkinetic extraocular muscle movements share findings that are consistent with the neurogenic type of muscle atrophy. This result suggests a common underlying etiology with variable clinical findings, representing the ocular counterpart of congenital cranial dysinnervation disorder, which has been reported as ocular congenital cranial dysinnervation disorder.

## INTRODUCTION

The Marcus Gunn phenomenon (MGP) is a form of congenital ptosis characterized by synkinetic movement of the eyelid with movement of the jaw. The abnormal movement representing synkinesis may be caused by abnormal connections between the trigeminal nerve and the upper division of the third cranial nerve.

A study conducted in 1988 by Lyness et al.^(1)^ demonstrated numerous histological changes in the muscle fibers of patients with MGP compared with that in controls. In samples collected from patients with MGP, they identified loss of fibers, atrophy, compensatory hypertrophy of remaining fibers, and changes in mitochondrial distribution within the fibers. They also identified changes in both eyelids, and not only the clinically affected side. These changes were consistent with changes in muscle fibers caused by a neurogenic etiology.

Joyce et al. published a detailed review on the histopathological differences of muscle fiber atrophy features between cases caused by muscular atrophy and those caused by neurogenic atrophy^(2)^. Their findings were consistent with the findings of MGP outlined by Lyness et al. They emphasized the use of modified Gomori trichrome staining for identifying mitochondrial abnormalities among other changes. Ehmsen and Hoke^(3)^ also summarized the histological features of skeletal muscle changes associated with neurogenic atrophy.

We propose that these changes can be found in both MGP and other similar conditions of eyelid misfiring, thereby suggesting a shared pathophysiology as an ocular subtype of congenital cranial dysinnervation disorder (oCCDD). Therefore, we conducted this study to perform a retrospective histopathological analysis of samples of patients with MGP and other misfiring conditions using modified Gomori trichrome staining. Further histopathological and genetic studies of these samples may yield additional information of diagnostic and etiological value.

## METHODS

This was a retrospective histopathological analysis of MGP biopsy samples conducted between January 1, 2010, and April 30, 2022. The patient population consisted of all pediatric patients with a diagnosis of congenital ptosis, MGP, or synkinetic extraocular muscle (EOM) movements indicating misfiring that involved the upper eyelids. The inclusion criteria were patients with ptosis and a diagnosis of MGP and patients with misfiring similar to MGP. The exclusion criteria were patients who did not undergo surgical repair/biopsy and patients with insufficient muscle tissue in the biopsied samples. We first identified all cases of ptosis that were operated during the study period and then extracted cases with typical MGP or similar ptosis with misfiring according to the abovementioned inclusion criteria.

All samples have undergone routine staining (hematoxylin and eosin/periodic acid-Schiff), and special modified Gomori trichrome staining that was added only for the included cases. Then, the histopathological features of the cases were identified by two experienced ocular pathologists. The corresponding clinical data were also collected, which included age at the time of surgery, demographic information, assessment of ptosis, family history, and surgical information. Correlation of the staining properties in nine samples of MGP cases was compared with two similar cases of ptosis with different clinical patterns of misfiring. A written general informed consent was obtained from all participants and/or their legal guardians for the publication of identifying information/images in an online open-access publication. The corresponding author will act as a guarantor and correspond with the journal from this point onward and in case the data are requested. This study does not involve drug trials or human transplantation.

## RESULTS

A total of 136 patients with congenital ptosis were identified in this study, among whom, 11 (8%) had eyelid misfiring, 9 (6.6%) had MGP, and 2 (1.4%) had upper eyelid ptosis and muscle synkinetic misfiring with EOM eye movement mimicking MGP. All the 11 patients with eyelid misfiring underwent surgical repair for ptosis in one or both eyelids. Among the nine patients with MGP, one patient had bilateral ptosis, and the remaining eight patients had equal distribution between the right and left sides. The female-to-male ratio was 7:2. Clinically, the severity of ptosis was variable from poor to excellent among patients with MGP, and the marginal reflex distance was 0-2 mm. The age at the time of the primary surgical procedure was 3-13 years with a median age of 9 years. Levator resection was the most common surgical treatment for ptosis with MGP in five patients, whereas the other four patients underwent removal of a portion of the levator muscle above the Whitnall’s ligament through an eyelid crease incision with a frontalis sling. Four patients required a second repair procedure for residual ptosis in the same eyelid. The average follow-up period was 5.2 (range 1-13) years. The overall outcome in patients with MGP was satisfactory. Table 1 presents a summary of these cases.

**Table 1:** Summary of the9nine cases of ptosis with Marcus Gunn jaw-winking phenomenon included in the histopathological analysis

Case number	Gender	Age at first surgery	First surgery/side	MRD Pre-op	LF Pre-op	FU Duration	Outcome (MRD)/ additional surgery
1	Female	6 years	Levator disinsertion + frontalis sling/right UL	-2	Good (10 mm)	5 years	Satisfactory (1 mm) after frontalis sling revision 1 month after the first surgery
2	Female	5 years	5-mm levator resection/left UL	1	Fair (7 mm)	7 years	Satisfactory (2 mm) after revision of levator resection 2 months after the first surgery
3^**^	Male	3 years	25-mm levator resection/right UL	<0	Poor (4 mm)	7 years	Satisfactory (2 mm) after levator disinsertion + frontalis sling 2 years after the first surgery
4	Female	9 years	17-mm levator resection/left UL	1	Good (8 mm)	3 years	Satisfactory (2.5 mm) after single surgery
5	Female	10 years	Levator disinsertion + frontalis sling^*^/left UL	0.5	Excellent (15 mm)	13 years	Satisfactory (3 mm) after single surgery
6	Female	13 years	Levator disinsertion + frontalis sling/left UL	2	Excellent (13 mm)	11 years	Satisfactory (3 mm) after repeated frontalis sling 5 years after the first surgery
7	Female	10 years	10-mm levator resection^*^/right UL	0	Excellent (12 mm)	4 years	Satisfactory (3 mm) after single surgery
8	Male	12 years	16-mm levator resection/right UL	1	Good (11 mm)	2 years	Satisfactory (2 mm) after single surgery
9	Female	8 years	Levator disinsertion + frontalis sling/right UL	2	Poor (3 mm)	3 years	Satisfactory (4 mm) after single surgery

UL= Upper lid; MRD= Marginal reflex distance; LF= Levator function; Pre-op= Preoperative; FU= Follow-up

* Cases 5 and 7 underwent UL crease formation on the same side 15 and 2 years after surgery, respectively.

** Case 3 had bilateral ptosis, worse on the operated side (right UL).

**Figure 1:**
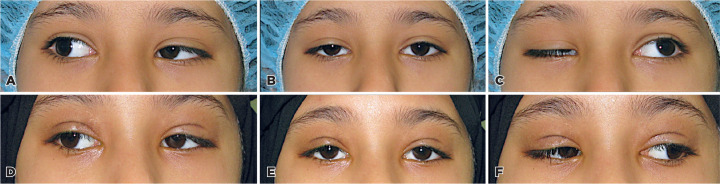
Preoperative appearance of one of the patients with synkinesis showing bilateral ptosis on adduction of the eye and subsequent retraction with abduction when looking straight in A, to the right in B, and left in C. The corresponding improvement in her appearance with the same directions of gaze in D, E, and F.

We found two unique cases of misfiring in two sisters, both of whom had bilateral ptosis on adduction of the eye. The first patient, a 7-year-old girl, had bilateral ptosis on adduction and retraction on abduction (Figure 1). She received a bilateral frontalis flap and then a surgical revision after 2 years. The second patient underwent surgical repair at the age of 11 years. She underwent a bilateral frontalis flap and two subsequent surgical revisions for ptosis.

**Figure 2:**
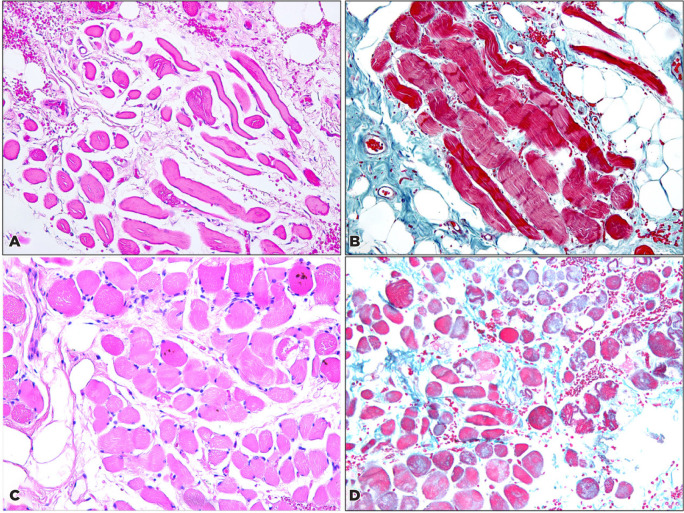
(A). Levator muscle fibers in a case of typical MGP showing variation in the diameter of the separated muscle fibers (original magnification ×200 hematoxylin and eosin staining). (B). Appearance of the uneven staining with clumping and typical ragged red fibers observed in neurogenic muscle atrophy from the same specimen (original magnification ×200 Gomori trichrome staining). (C). Muscle sample collected from the levator muscle of one of the two patients with synkinesis similarly showing atrophy and separation of the muscle fibers (original magnification ×200 hematoxylin and eosin staining). (D). Sparse muscle fibers with an irregular staining pattern in the same specimen were evident using the special stain used for MGP cases (original magnification ×200 Gomori trichrome staining).

Histopathological analysis of the excised muscle and tendon samples showed that all samples of levator muscle and tendon exhibited overall loss of muscle fibers, irregular-modified Gomori trichrome staining with clumping suggestive of the accumulation of mitochondria in the center of individual fibers, and atrophy of muscle fibers. An example of these findings in a MGP sample is depicted in Figure 2 A, B. The histopathological sample collected from one of the patients with ptosis and misfiring of the eyelid with adduction of the globe is presented in Figure 2 C, D.

## DISCUSSION

The overall prevalence of MGP and eyelid misfiring was 8% among patients diagnosed with congenital ptosis who had undergone surgical repair in our cohort. A previous study reported an MGP prevalence of 2%-13% among patients diagnosed with congenital ptosis^(4)^. In another study, there were 72 patients with MGP among 848 cases of congenital ptosis (constituting 8.5%), which is similar to our results. That study also reported that the involvement of either side to be affected was equal, and 20% of cases involved both eyelids^(5)^. Other studies have described a near-equal distribution of MGP laterality with a slight predominance of left eye involvement^(6^,^7)^. Furthermore, other individual case reports have described bilateral MGP, and among our cases, only one patient had bilateral MGP^(8^,^9)^.

MGP typically presents with ptosis and synkinetic movement of the eyelid with voluntary movement of the jaw, but it may also occur without ptosis^(5)^. Although it is believed that MGP occurs as a congenital phenomenon, it has also been reported as an acquired condition in two cases. The first case was attributed to a cricket ball injury, and the second occurred after a trauma during delivery^(6)^. This onset may be attributed to an incidental finding after the trauma, which emphasizes the complexity of MGP and the need to explore and understand the underlying etiology to improve our approach and management methods.

Theories behind the misfiring observed in patients with MGP include an abnormal connection between the trigeminal and third cranial nerve, resulting in involuntary movement of eyelids with voluntary movement of the jaw or disinhibition of a primitive reflex. MGP has also been reported to be associated with ipsilateral upper eyelid myokymia, which suggests the complexity of the faulty innervation that might manifest in these cases and supports a common etiology of these clinically variable misfiring conditions as demonstrated in our study^(10)^.

We observed a varying range of surgical outcomes in MGP. Four patients with MGP required additional surgeries for residual and recurrent ptosis. In a previous report, 9 patients with MGP who underwent levator resection had residual ptosis^(11)^. We found that levator resection was the most common primary procedure, and frontalis sling was the most common secondary procedure for residual ptosis. Al-Essa et al. reported a 70% recurrence rate of ptosis after levator resection in patients with MGP^(12)^.

There have been several studies on histopathological changes in nonocular muscle biopsies in cases of myopathies to differentiate between dystrophic muscle changes and neurogenic disorders, especially when associated with an inflammatory etiology. These studies primarily evaluated muscle fiber atrophy/degeneration, fibrosis, nuclear changes and inclusions within muscle fibers, fatty infiltration, regenerating fibers, and inflammatory infiltrate^(13^,^14)^. In one of those studies, the authors used the modified Gomori trichrome staining to demonstrate the difference between polygonal shaped multinucleated normal muscle fibers with minimal connective tissue and the fascicular atrophic fibers in neurogenic disorders^(13)^. However, they concluded that histopathological changes are not specific for each muscle disorder and that diagnosis should be aided by clinical evaluation and genetic testing^(13^-^15)^. In our study, we observed histopathological changes consistent with a neurogenic type of atrophy in all muscle fibers. These changes included the overall loss of muscle fibers, clumped uneven staining of muscle fibers with modified Gomori trichrome, atrophy of muscle fibers, and secondary compensatory hypertrophy of fibers. These changes were identified in the MGP samples as well as the samples collected from the four upper eyelids of the two patients who had synkinesis with synergetic extraocular muscle movements and were both operated bilaterally. These characteristics have also been described in other nonophthalmic skeletal muscles affected by neurogenic atrophy^(3)^. Therefore, these characteristics would further support an underlying shared neurogenic etiology in all our cases.

A proper histopathological analysis of biopsied samples requires a sufficient muscle portion in the surgically excised samples. The length of the excised levator aponeurosis can often be limited to the tendinous segment of the levator, excluding the muscle fibers that are essential for the analysis. This can be a limiting factor for the complete histopathological analysis of the skeletal muscle fiber. Furthermore, the rarity of the disease and varying severities have contributed to an overall smaller population size who have undergone repair and subsequent histopathological analysis in ocular cases. In histopathological studies of the levator muscle in congenital ptosis, atrophy of muscle fibers, centralization of nuclei, and hyalinization of cytoplasm have also been demonstrated; however, more significant variation in the size of muscle fibers has been detected in MGP cases favoring dysgenesis etiology^(16^,^17)^.

We found two sisters with abnormal miswiring resulting in bilateral ptosis and retraction of the eyelids with adduction and abduction of the eyes, respectively, representing a form of oCCDD in a similar manner to MGP cases. A recent study of oCCDD by Jurgens et al. indicated the heterogeneity of these disorders either in isolation or as a part of syndromic phenotype^(15)^. Therefore, underlying genetic risk factors with potential for inheritance may aid in the diagnosis. Further genetic testing might be essential to help understand this phenomenon because cases of familial MGP have been described previously^(18^-^20)^. Further studies of particular genes in oCCDD, such as *MYH10* and *TGF-*beta, have been recommended^(15)^. Other studies have found a *KIF21A* novel mutation linked to congenital fibrosis of extraocular muscles with MGP^(21^-^22)^.

In conclusion, our histopathological analysis of samples of MGP and the other two patients with miswiring conditions demonstrated changes consistent with those described previously in the neurogenic atrophy of muscle fibers collected from ocular and nonocular sites. The histopathological changes between MGP samples and those of patients with synkinesis were comparable, indicating heterogeneous forms of oCCDD. Considering the lack of a clear understanding of the underlying mechanism and the possible genetic etiology of MGP and other forms of oCCDD, further studies are required to help understand these complex entities.
